# Note upon Odontology

**Published:** 1859-07

**Authors:** Christopher Johnston


### ARTICLE VI.

*Note upon Odontology.* By CHRISTOPHER JOHNSTON, M. D.

WHOEVER takes the trouble to compare an ancient with a recent treatise upon geology, finds himself much perplexed to reconcile the differing nomenclature; and it is not until he has fully comprehended the broad and certain basis of modern geology, and realized the vast, important, nay indispensable aid which palæontology affords in the identification, the separation, and the grouping together of, perhaps, every particular stratum composing the earth's crust, that he becomes aware in how grand a manner a great subject is treated by modern scientists. In former times, there prevailed, in every department of learning, systems, theories and classifications which waxed and waned, and were supplanted by other speculative substitutes for truth. In medicine, for example, the tenets of Galen ruled physicians for centuries, and were maintained in the schools, even when in opposition to observed phenomena of nature. It was enough to affirm "*Dixit Galenus*" to overwhelm the laborious and patient investigator, who must henceforth content himself with viewing everything in the glimmer of artificial light. In geology, there resulted from a comparison of human works with those of the Divine Artificer, certain assumptions which presently came to be regarded as tests of the purity of geologic doctrine. Without taking into consideration the changes inevitable in rocks or groups of rocks, an immutable basis of primary strata was established, and on these the aqueous and volcanic rocks were set as sequence in fact and in point of time.

This theory, now nearly obsolete, gave way before the power of a higher reason, which dawned about a century ago, when Lehman, dismissing the idea of foundation and superstructure, announced a "bold generalization," which referred certain rocks to the period preceding the existence of created beings, and others to those epochs contemporaneous with, and following, their appearance upon the earth.

"In his primitive class, he said, such as granite and gneiss, there are *no organic remains*, nor any signs of materials derived from the ruin of pre-existing rocks. Their origin, therefore, may have been purely chemical, antecedent to the creation of living beings, and probably, coeval with the birth of the world itself. The secondary formations, on the contrary, which often contain sand, pebbles, and *organic remains*, must have been mechanical deposits, produced after the planet had become the habitation of plants and animals."*

The investigation of facts has measurably replaced the study of theories; and the geological problem of nowadays involves three questions, namely, *superposition*, *mineral composition*, and *organic remains*, being an addition of two elements to the single proposition of ancient writers upon

* **Lyell, Elements of Geology.**

the formation and precedence of the earth's strata. Therefore, every work upon geology must embrace these considerations, at the least; and while the field of inquiry is greatly extended, the varied sources of information render knowledge more certain, more precise, more massive.

Palæontology, or the science which treats of fossil remains, both animal and vegetable, is not to be understood as simply determining the structure and probable form and appearance of things which once had life. It goes farther than this—the characters that derive from it become associated with the age of a formation, and constitute a criterion of the highest importance in the comparison of contemporaneous strata, and the distinction of strata, the dates of origin of which are separated by an interval of lesser or greater extent. The study of palæontology, consequently, necessitates, and almost presupposes an acquaintance with geology; for however interesting the fact that such an animal or such a plant was invested with a particular form, agreeable or hideous, it is almost valueless in science, unless the surroundings are also known, such as the circumstances under which it existed, as the climate, the part of the globe, the habits, whether of mountain or valley, of lake, river or sea, and what is equally consequential, the *age* of the formation upon which it appeared, flourished, and finally become entombed.

It oftentimes happens that the conditions under which an animal has been buried in soil were unfavorable for the preservation of even those parts of the body which usually resist decay; so that it behooves the palæontologist to familiarize himself with the characters of minutest recognizable portions that have escaped decomposition. But it is a convertible proposition that to know what is new, one must know what is old—therefore, to know whether an organism belonged to a pre-Adamic period, an acquaintance with the natural history of the human period is indispensable; and not only that, without a complete knowledge of the compared anatomy of existing genera and species, it would be impossible to refer fragmentary integrals of a lost being to their true position in the economy, as well as to assign the creature a proper place in the animated scale. Suppose, for instance, that a broken bone be disinterred, or lay exposed to the light by the falling in of the earth of a river's bank—the first question that arises is to what animal did that bone belong? The comparative anatomist, knowing what is, recognizes the fragment as a portion of the skeleton of a horse. But a greater difficulty now arises; was the living possessor of that bone a representative of an ancient type, or did he, as a member of a recent species, roam the plains from which civilization has driven his wild fellows, or on which it has yoked them to the plow. Comparative anatomy, pelæontology and geology, must tell in what age, and upon what stratum of the earth's crust he stamped the hoof.

What we here suppose for illustration, did actually occur. In 1855 Prof. F. S. Holmes, of Charleston, S. C., collected on Ashley river, from *post-pliocene* beds, (the first removed in antiquity from the new estor *recent* strata,) a great number of fossil bones. These were recognized as remains of the horse, hog, sheep, dog and ox; bison, tapir, peccary, beaver, muskrat and elk; mastodon, megatherium, megalonyx, glyptodon, mylodon and hipparion. In such company could the horse be claimed as recent? or had horse, ox and sheep become accidental occupants of the same beds with true post-pliocene and eocene fossils? Now there occurred scattered among these bones, *teeth* of all, or nearly all the animals named. These organs, the hardest and most enduring parts of the animal, are not unfrequently the sole remaining traces of creatures which the post has swallowed up; and their distinctive number, form, structure, mode of growth, and succession, all harmonizing with the various functions assigned to them in different classes, orders and genera, and with the modifications concomitant with specific differences of habit. So positive are the indications afforded by the teeth that not only may the animal once possessing it be named with certainty mammal, reptile or fish, but be identified as horse, mastodon, elephant, saurian, batrachian, shark or ray; its age, its species, may be ascertained, and even the nature of its food and its habits. It is not, therefore, surprising that odontology, as a chief department of comparative anatomy, should have borne a notable part in the investigation of the post-pliocene fossils of Ashley river.

No country offers the odontologist a richer field than our own America, older, geologically, than "old" Europe. In every quarter, the disemboweled earth yields up its fossil treasures—here of mammal, there of fishes; and in the Cretaceous, east, south and west, a profusion of reptilian remains. Among the most remarkable of these last are the teeth of a thecodont saurian, which we have named Astrodon, found near Bladensburg; the magnificent teeth, jaw and humerus of *Mosasaurus* discovered near Wilmington, Del.; and the colossal bones and exquisitely beautiful teeth of *Hadrosaurus*, an extinct lizard. This huge herbivorous saurian, allied to the famed *Iguanodon* of the Wealden England, was about twenty-five feet in length. Its *humerus* is twenty-three inches long, and seven inches broad at the tuberosities; the *ulna* twenty-three inches long and seven inches in circumference at the middle. The *femur* is no less than forty inches in length, ten inches wide through the internal condyle, and seventeen inches in circumference midway in its length; and the *tibia* measured thirty-six and a half inches, the circumference being about twelve inches.

It was no difficult matter for a Leidy to recognize in this gigantic skeleton the framework of a lizard, and to affirm its high antiquity. But the diet of ancient as well as of existing lizards was various: some saurians feeding on insects, or birds, or flesh, in some form, while others are vegetable feeders, a circumstance to which the feet and jaws point vaguely, but which is established in the most positive manner by those organs having a first and very close relation with the aliment, the teeth. The tooth of Iguanodon found isolated in the Wealden by Mantell, was not recognized as saurian by Cuvier, who, nevertheless, in referring it to an extinct Rhinoceros, showed that its form, structure, and manner of wear, left no doubt as to the herbivorous character of its possessor. But he subsequently justified himself fully, and proclaimed the true affinities of the great extinct creature, declaring it to have been a herbivorous saurian.

Of Hadrosaurus a number of teeth were found, black, and well preserved; and conical on one side, the other being wrought into two lateral grooves, leaving a sharp longitudinal ridge in the middle, like the side of a bayonet. Certain data were furnished by these portions of the buccal armature as to the feeding habits of the great extinct saurian; and Hadrosaurus was, from all the indications, declared to be an amphibious and *herbivorous* lizard. We enjoyed the rare opportunity of making thin sections of one of the teeth, which we shared with Prof. Leidy; and certainly nothing could exceed the beauty of the microscopic structure, far surpassing that of the renowned Iguanodon, nor could any testimony be more conclusive as to the use to which the tooth must have been applied. It is typically herbivorous.

Every day is bringing new facts to science, and enlarging the mind of man to fit him for a more worshipful contemplation of the Creator in the grandeur and exhaustless fertility of design in His works. From the remotest periods a procession of organic forms advances slowly through the misty portals of time. As it proceeds, new things endowed with life and action join the throng, but old forms drop by the wayside, and appear no more forever, but as inanimate dust, marking the "foot-prints of the Creator." The great earth itself, is rent with commotions—a continent sinks below or rises above the mighty deep—but onward, still onward, grandly moves the pageant, evermore in the growing light. The ground trembles beneath its heavy tread—its pulse beats the "seconds of eternity," and its voices shake the air as awful shout goes up when the Almighty places a spark of His own immortality upon the brow of the noblest, the lordliest form of being, and with which Divinity itself will deign to be invested.

We swell the living crowd ; and shall we not learn from the pathway that has been trodden, whither we are tending?

